# Radioligand saturation binding for quantitative analysis of ligand-receptor interactions

**DOI:** 10.1007/s41048-016-0016-5

**Published:** 2016-02-14

**Authors:** Chengyan Dong, Zhaofei Liu, Fan Wang

**Affiliations:** 1 Interdisciplinary Laboratory, Institute of Biophysics, Chinese Academy of Sciences, Beijing, 100101 China; 2 Medical Isotopes Research Center and Department of Radiation Medicine, School of Basic Medical Sciences, Peking University, Beijing, 100191 China

**Keywords:** Equilibrium constant, Maximum density of receptors, Saturation binding assays, I-125 labeling, Radioligand

## Abstract

The reversible combination of a ligand with specific sites on the surface of a receptor is one of the most important processes in biochemistry. A classic equation with a useful simple graphical method was introduced to obtain the equilibrium constant, *K*_d_, and the maximum density of receptors, *B*_max_. The entire ^125^I-labeled ligand binding experiment includes three parts: the radiolabeling, cell saturation binding assays and the data analysis. The assay format described here is quick, simple, inexpensive, and effective, and provides a gold standard for the quantification of ligand-receptor interactions. Although the binding assays and quantitative analysis have not changed dramatically compared to the original methods, we integrate all the parts to calculate the parameters in one concise protocol and adjust many details according to our experience. In every step, several optional methods are provided to accommodate different experimental conditions. All these refinements make the whole protocol more understandable and user-friendly. In general, the experiment takes one person less than 8 h to complete, and the data analysis could be accomplished within 2 h.

## INTRODUCTION

Research on receptors has developed very quickly in the past few decades. The interactions between ligands and receptors generate and enhance signals for recognition, feedback and crosstalk in cells (Klotz 1985; Wilkinson 2004). Receptors are divided into two groups by their location: in the membrane or the nucleus. Hormones and transmitters can selectively recognize and bind to receptors to accomplish a biological process. Many drugs are designed and improved based on utilizing the ligand-receptor interaction.

Radioligand binding is widely used to define receptor function at the molecular level. The first radiolabeled binding assay was developed during the 1960s (Maguire et al. 2012). This radioligand binding assay (RBA) remains the most sensitive quantitative approach to measuring binding parameters in vitro, even in low receptor-expression cells (Rovati 1993; Keen 1995). Since the 1970s, the application of RBA has developed rapidly, with better receptor preparations, more radiolabeled ligands and higher radioactivity. Currently, appropriate ligands radiolabeled with tritium or iodine are available for the study of many receptors, including adrenergic, cholinergic, dopaminergic, serotonergic, and opiate receptors (Tallarida et al. 1988). This widespread availability has led to a rapid growth in the use of radioligand binding assays to characterize novel receptors and receptor subtypes and determine their anatomical distribution, and these assays play a vital role in the development of drugs by the pharmaceutical industry (Bylund and Toews 1993; Carpenter et al. 2002).

Unlabeled ligands require a radioactive isotope to be incorporated into the molecule. It is occasionally necessary to modify the structure of the ligands to provide a suitable site for radiolabeling. However, it is imperative that the selectivity and specificity of the ligand be retained after the modification and radiolabeling. Two basic parameters of this binding site can be studied by kinetic and saturation analysis: the affinity of the ligand for its recognition site, the *K*_d_, and an estimate of the number of binding sites in a given tissue, the *B*_max_ (Williams and Jacobson 1990). The *K*_d_ is the equilibrium dissociation constant, which is the concentration of ligand that will occupy 50% of the receptors. The generally accepted standard is that a ligand-receptor binding with a *K*_d_ of 1 nmol/L or less has a high affinity, whereas ligands binding with a *K*_d_ of 1 µmol/L or more have low affinity (Davenport and Russell 1996). *B*_max_ signifies the maximum density of receptors. This value is unique to a particular tissue in the binding assay, and it is usually corrected using the amount of protein or cells present.

## MECHANISM OF ACTION

Analysis of radioligand binding experiments is based on a simple model; the law of mass action that describes the interaction between one molecule of ligand and one receptor molecule. For example, a neurotransmitter binds to the synaptic receptor to initiate the neurobiological process, or an antibody binds to an antigen to initiate the immunological response. In the simplest and most common case, this is a bimolecular reaction between a ligand and a receptor. This model assumes that binding is reversible (Anderson 1994).

1 $$\left[ {\text{R}} \right] + \left[ {\text{L}} \right] \rightleftarrows \left[ {\text{RL}} \right],$$

where [R] is the concentration of free receptor, [L] is the concentration of free ligand, [RL] is the concentration of the complex, *k*_1_ is the association rate constant, and *k*_2_ is the dissociation rate constant,

2 $$K_{\text{d}} = \frac{{k_{2} }}{{k_{1} }} = \frac{{[{\text{R}}][{\text{L}}]}}{{ [ {\text{RL}}]}}.$$

The density of unbound receptors [R] and ligands [L] cannot be determined but could be given:

3 $$\left[ {\text{R}} \right] = \left[ {\text{RL}} \right] - \left[ {\text{RL}} \right].$$

Hence, Eq. 2 could be rearranged to:

4 $$\frac{{[{\text{RL}}]}}{{ [ {\text{L}}]}} = \frac{{[{\text{RT}}]}}{{K_{\text{d}} }} - \frac{{[{\text{RL}}]}}{{K_{\text{d}} }}.$$

For further analysis, [SB] could represent the concentration of ligand bound to the receptor, [F] represents the concentration of unbound ligand or “free” ligand, and *B*_max_ represents the greatest attainable concentration of bound ligand:

5 $$\frac{{[{\text{SB}}]}}{{ [ {\text{F}}]}} = \frac{{B_{\hbox{max} } }}{{K_{\text{d}} }} - \frac{{[{\text{RL}}]}}{{K_{\text{d}} }}.$$

Thus, a plot of fractional [SB]/[F] vs. [RL] will give the basic information of the ligand-receptor interactions, known as a “Scatchard plot”. An alternative is the Woolf plot, a plot of fractional [F]/[SB] vs. [L]:

6 $$\frac{{[{\text{F}}]}}{{[{\text{SB}}]}} = \frac{{K_{\text{d}} }}{{B_{\hbox{max} } }} + \frac{{[{\text{L}}]}}{{B_{\hbox{max} } }}.$$

## APPLICATIONS AND LIMITATIONS

Radioligand-binding techniques are applicable to any receptor of interest, provided it has a relative ligand that could selectively bind the receptor and be labeled with radioactive isotopes. Antibodies, proteins and peptides that contain Tyr could easily be labeled with iodine. Radioligands provide precise probes to quantify the initial interaction between ligands and receptors. For example, the kinetics of the association and dissociation of radioligands can be accurately examined from a simple tissue expressing the specific target receptors. A series of concentrations of unlabeled ligands could inhibit the forces established between radioligands and receptors, which could be used to measure the equilibrium dissociation constants. Radioligand binding also permits a characterization of receptor subtypes with different affinities and provides an estimate of their relative proportions, especially in the study of central nervous system receptors, where the effects of neurotransmitters are complex, and isolated tissue preparations are unfeasible (Tallarida et al. 1988). Radioligand binding assays can also be used to monitor the changes in receptor density, perhaps resulting from the pathological conditions of pharmacological intervention. Furthermore, the Scatchard plot is a useful diagnostic tool to determine whether more than one ligand molecules bind to a single receptor (Hollemans and Bertina 1975; Rovati 1998). A concave upward plot is indicative of nonspecific binding, negative cooperativity, or multiple classes of binding sites. A concave downward plot suggests either positive cooperativity or instability of the ligand (Wilkinson 2004).

However, binding parameters could be affected by many factors including the specific radioactivity, the type and ionic strength of the buffer, the presence of divalent ions and the temperature. The results are not sufficient to reflect the real physiological response mediated by the receptor in this homogenate preparation, such as minimizing degradation of the ligand (Davenport and Russell 1996). In addition, radioligand-binding assays cannot adequately discriminate between full agonists that elicit maximal physiological responses and partial agonists that cannot elicit a maximal response (Tallarida et al. 1988).

## SUMMARIZED PROCEDURE

1. Dissolve iodogen in chloroform at a concentration of 2 mg/mL, evaporate the chloroform and make the iodogen-coated tube under an N_2_ stream.
2. Dissolve Protein L in 0.2 mol/L phosphate buffer (pH 7.4) at a concentration of ∼2 mg/mL.
3. Mix 50 µL Protein L, 50 µL PB (0.2 mol/L, pH 7.4) and 50–100 µL Na^125^I (>40 MBq) in an iodegen-coated tube. Incubate for 7–8 min at room temperature.
4. Remove the reaction mixture from the iodogen tube and purify the radiolabeled protein by size exclusion chromatography using a PD MidiTrap G-25 column.
5. Count the activity in the final recovered tube and calculate the specific activity.
6. Wash two 96-well plates with pre-cooled cell-binding buffer for three times (100 µL each well) and use the vacuum manifold to remove the buffer. Place two 96-well plates for specific binding and non-specific binding respectively.
7. From a stock of two million receptor-overexpressed cells per mL of cell-binding buffer (total volume > 1.5 mL), add 1 × 10^5^ cells (50 µL) each well in the 96-well plate.
8. Prepare three stock solutions of different concentration ^125^I-Protein L (e.g., 0.1, 1 and 10 µg/mL) in cell-binding buffer.
9. Add the cells and ^125^I labeled ligand into the plates according to calculation of final concentration for each well (*N* = 4), and adjust the total volume to 200 µL per well with cell-binding buffer and incubate for 2 h at 4 °C.
10. Use the vacuum manifold to remove the incubation buffer from the plates and wash 5–10 times with cell-binding buffer (100 µL/well).
11. Heat-dry the plates in the dry bath incubator, and collect the membrane from each well into polystyrene culture test tubes.
12. Add 4–7 tubes of standard samples to measure. Then measure the radioactivity on each membrane with a γ-counter.
13. For each experimental measurement, subtract the cpm values of groups. Added activities [TA], total binding activities [TB] and non-specific binding activities [NSB] could be measured and calculated by the activities on the membranes of plates.
14. Calculate [SB], [LT], [RL], [L] and [F] using these corrected values.
15. *K*_d_ value and *B*_max_ could be calculated by Scatchard Plot, Woolf Plot or the software.

## PROCEDURE

### Radiolabeled protein preparation [TIMING] ~1 h

1. There are two options for labeling the proteins.
  (A) **Option A: Chloramine-T method** (Hunter 1970; Opresko et al. 1980).
  i. Dissolve Protein L (see “Reagent setup” section) in 0.2 mol/L phosphate buffer (pH 7.4) at a concentration of 2 mg/mL.
  ii. Mix the following reagents: 50 µL Protein L, 50–100 µL Na^125^I (>40 MBq) and 100 µg Chloramine-T (1 mg/mL in 100 µL 0.2 mol/L PB, pH 7.4). Incubate for 40 s at room temperature.
    [**CRITICAL STEP]** It is highly recommended to limit the reaction time in 1–3 min.
  iii. Add 100 µL Na_2_S_2_O_5_ (200 µg in ddH_2_O) and 100 µL 1% KI (1 mg in ddH_2_O) to the mixture.
  (B) **Option B: Iodogen method** (Bailey 1996).
  i. Dissolve Protein L (see “Reagent setup” section) in 0.2 mol/L phosphate buffer (pH 7.4) at a concentration of ∼2 mg/mL.
  ii. Dissolve iodogen in chloroform at a concentration of 2 mg/mL. Transfer aliquots of 25 µL (50 µg) to a glass-bottomed screw cap vial. Evaporate the chloroform to dryness under an N_2_ stream, leaving a thin coating of iodogen in the tube. Store the desiccated iodogen-coated tubes at −20 °C until required for iodination.
  iii. Mix 50 µL Protein L, 50 µL PB (0.2 mol/L, pH 7.4) and 50–100 µL Na^125^I (>40 MBq) in an iodogen-coated tube. Incubate for 7–8 min at room temperature.
    [**CRITICAL STEP]** It is highly recommended to maintain the duration of the reaction between 5–10 min.
  iv. Remove the reaction mixture from the iodogen tube and apply to purifying.
**[CAUTION!]** It is imperative to obtain appropriate training from the institutional radiation safety office before experimenting with radioactivity. Abide by all relevant regulatory rules and use appropriate protection when handling radioactivity. Dispose of the ^125^I-containing radioactive waste according to the institutional radioactive waste disposal guidelines.
2. Purification: Purify the radiolabeled protein by size exclusion chromatography using a PD MidiTrap G-25 column.
  (A) Preparation and equilibration: Remove the caps and the storage solution. Fill the column with PBS and discard the flow-through. Repeat this procedure twice (three times in total).
  (B) Sample application: Add a maximum of 1.0 mL of sample to the column. Apply the sample slowly in the middle of the packed bed and discard the flow through.
  (C) Elution: Place a clean tube for sample collection under the column. Elute with 1.5 mL PBS and collect the products.
3. Count the activity in the final recovered tube. The specific activity is calculated as the quotient between the recovered activity and the total amount of protein. This calculation assumes that 100% of the protein added to the iodination was recovered, which is not typical. (Analytical Techniques, T.P. Mommson)
  $${\text{Specific activity}} = \frac{{\text{Radioactivity}}}{{\text{Protein mass}}} {\text{(Ci/g)}}.$$
**[CRITICAL STEP]** To obtain an optimal result, it is sufficient to utilize radioligands with high specific activity (>20 Ci/mmol).

### **Saturation binding[TIMING]** 5–7 h

4 Wash two 96-well plates with pre-cooled cell-binding buffer three times (100 µL each well) and use the vacuum manifold to remove the buffer. One 96-well plate (Plate A) will be used for specific binding and the other one (Plate B) will be used for non-specific binding(Cai and Chen 2008).
5 From a stock of two million receptor-overexpressed cells per mL of cell-binding buffer (total volume > 1.5 mL), add 1 × 10^5^ cells (50 µL) to each well in the 96-well plate.
6 Prepare three stock solutions of different concentrations of ^125^I-Protein L (e.g., 0.1, 1 and 10 µg/mL) in cell-binding buffer. Typically a series of concentrations between 1 ng/200 µL and 1 µg/200 µL will be needed per well.
**[CAUTION!]** It is imperative to obtain the appropriate preparatory training and abide by all regulatory rules when handling radioactivity.
**[? TROUBLESHOOTING]**
7 Add the cells and ^125^I-labeled ligand into Plate A following Table 1, and adjust the total volume to 200 µL per well with cell-binding buffer and incubate for 2 h at 4 ºC. More than four samples are recommended for each concentration.

**Table 1:** Sample adding strategy in the typical 96-well plate for the specific binding assay

Specific binding group												
No.	1	2	3	4	5	6	7	8	9	10	11	12
^125^I-Protein L (μg/mL)	0.1	0.1	0.1	0.1	1	1	1	1	10	10	10	10
^125^I-Protein L (μL)	10	20	50	80	10	20	50	80	10	20	50	80
Binding buffer (μL)	140	130	100	70	140	130	100	70	140	130	100	70
Cell solution (μL)	50	50	50	50	50	50	50	50	50	50	50	50
Total volume (μL)	200	200	200	200	200	200	200	200	200	200	200	200
**[? TROUBLESHOOTING]**
8 Add the cells, ^125^I-labeled ligands and excess cold Protein L into Plate B following Table 2, and adjust the total volume to 200 µL per well with cell-binding buffer and incubate for 2 h at 4 ºC as the last step. More than four samples are recommended for each concentration.

**Table 2:** Sample adding strategy in the typical 96-well plate for the non-specific binding assay

Non-specific binding group												
No.	1	2	3	4	5	6	7	8	9	10	11	12
^125^I-Protein L (μg/mL)	0.1	0.1	0.1	0.1	1	1	1	1	10	10	10	10
^125^I-Protein L (μL)	10	20	50	80	10	20	50	80	10	20	50	80
Cold Protein L (μL)	50	50	50	50	50	50	50	50	50	50	50	50
Binding buffer (μL)	90	80	50	20	90	80	50	20	90	80	50	20
Cell solution (μL)	50	50	50	50	50	50	50	50	50	50	50	50
Total volume (μL)	200	200	200	200	200	200	200	200	200	200	200	200
**[? TROUBLESHOOTING]**
9 Use the vacuum manifold to remove the incubation buffer from the 96-well plate and wash 5–10 times with cell-binding buffer (100 µL per well).
10 Heat-dry the 96-well plates in a dry bath incubator until all filter membranes are dry. This usually takes approximately 15 min.
11 Collect the membrane from each well into polystyrene culture test tubes.
12 Add 4–7 tubes of radiolabeled samples (standard samples) to measure.
**[PAUSE POINT]** The radioactivity on each membrane can be measured later, because ^125^I has a half-life of 60 d.
13 Measure the radioactivity on each membrane with a γ-counter.

### **Analysis[TIMING]** 1–2 h

14 For each experimental measurement, subtract the cpm values of the groups. Added activities [TA] could be measured using the standard samples. Total binding activities [TB] and non-specific binding activities [NSB] could be measured by the activities on the membranes of Plate A and B, respectively.
15 Using Eqs. (5)–(9), calculate [SB], [LT], [RL], [L] and [F] using these corrected values:
  7 $$\left[ {\text{SB}} \right] = \left[ {\text{TB}} \right] - \left[ {\text{NSB}} \right],$$
  8 $$[{\text{LT}}] = \frac{{[{\text{TA}}]({\text{cpm}})}}{{E\% \times [{\text{SA}}]({\upmu} {\text{Ci}}/{\text{nmol}}) \times 2.22 \times 10^{6} }} \times \frac{{10^{3} }}{{{\text{Volume }}({\text{L}})}}\,\left( {{\text{pmol}}/{\text{L}}} \right),$$
  9 $$[{\text{RL}}] = \frac{{[{\text{SB}}]({\text{cpm}})}}{{E\% \times [{\text{SA}}]({\upmu} {\text{Ci}}/{\text{nmol}}) \times 2.22 \times 10^{6} }} \times \frac{{10^{3} }}{{{\text{Volume }}({\text{L}})}}\,\left( {{\text{pmol}}/{\text{L}}} \right),$$
  10 $$\left[ {\text{L}} \right] = \left[ {\text{LT}} \right] - \left[ {\text{RL}} \right],$$
  11 $$\left[ {\text{F}} \right] = \left[ {\text{TA}} \right] - \left[ {\text{SB}} \right].$$
16 *K*_d_ and *B*_max_ could be calculated using a Scatchard plot, Woolf plot or the software.
  (A) **Option A: Scatchard plot**
  i. For each point on the concentration, enter [RL] into the *X* column and the value of [SB]/[F] into the *Y* column:
    12 $$\frac{{[{\text{SB}}]}}{{[{\text{F}}]}} = \frac{{B_{\hbox{max} } }}{{K_{\text{d}} }} - \frac{{[{\text{RL}}]}}{{K_{\text{d}} }}.$$
  ii. All the points are plotted and then linear regression is used to produce the line.
  iii. Referring to the Results sheet for the regression analysis, the *X*- and *Y*-axis intercepts could be calculated. The *X*-intercept represents the *B*_max_, and the *Y*-intercept represents *B*_max_/*K*_d_.
  (B) **Option B: Woolf plot**
  i. For each point on the concentration, enter [L] into the *X* column and the value of [F]/[SB] into the *Y* column:
    13 $$\frac{{ [ {\text{F}}]}}{{[{\text{SB}}]}} = \frac{{K_{\text{d}} }}{{B_{\hbox{max} } }} + \frac{{[{\text{L}}]}}{{B_{\hbox{max} } }}.$$
  ii. All the points are plotted and then linear regression is used to produce the line.
  iii. Referring to the results sheet for the regression analysis, the *X*- and *Y*-axis intercepts could be calculated. The *X*-intercept represents the *K*_d_, and the *Y*-intercept represents *K*_d_/*B*_max_.
  (C) **Option C: Saturation binding curve**
  i. Create a new project (file) on Prism(Motulsky 1996). For each point on the saturation-binding curve, enter the concentration of ligand into the *X* column and [SB] into the *Y* column.
  ii. Select “One site binding” under the “Nonlinear Regression dialog” box to analyze the data and produce a binding curve.
  iii. Prism displays the best-fit values (*B*_max_ and *K*_d_) for the binding parameters in the results sheets.

**[TIMING]**

Step 1–3: Preparation of the ^125^I-Protein L and cold ligands takes approximately 1 h.
Step 4–6: Preparation of the receptor samples takes approximately 1 h.
Step 7–13: The cell-binding assay usually takes 4–6 h, depending on how many samples are used.
Step 13–16: Activity measurements and data analyses take approximately 1–2 h.

**[? TROUBLESHOOTING]**

Step 6: The presence of certain metal ions (e.g., Mn^2+^ and Mg^2+^) in the cell-binding buffer is essential for receptor binding. Binding buffer without these ions will result in low counts from the collected membrane.
Step 7: It is necessary to add the cells and buffer with multiple-channel pipettes to reduce the time of this step. It takes practice to become skilled at adding serial concentrations of radioligand and cold ligand. It is important to stay focused and patient.
Step 8: The non-specific binding assay requires a large quantity of the cold ligand. Usually the ligands are difficult to prepare or very expensive. The Scatchard plot and the Woolf plot could be completed using fewer concentrations and fewer parallel samples. In total, about 20 samples are sufficient for fitting the linear regression.

## ANTICIPATED RESULTS

**Fig. 1:**
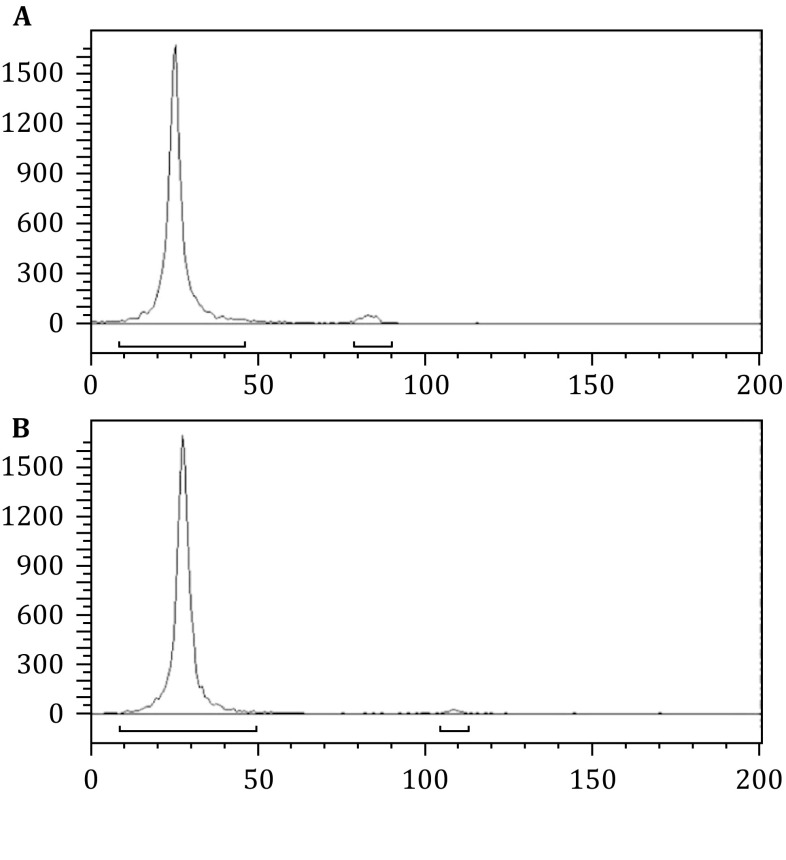
ITLC analysis of ^125^I-labeled Nimotuzumab. The labeling yield of ^125^I-Nimotuzumab was 97.6%, and the radiochemical purity was 98.5% after purification

**Fig. 2:**
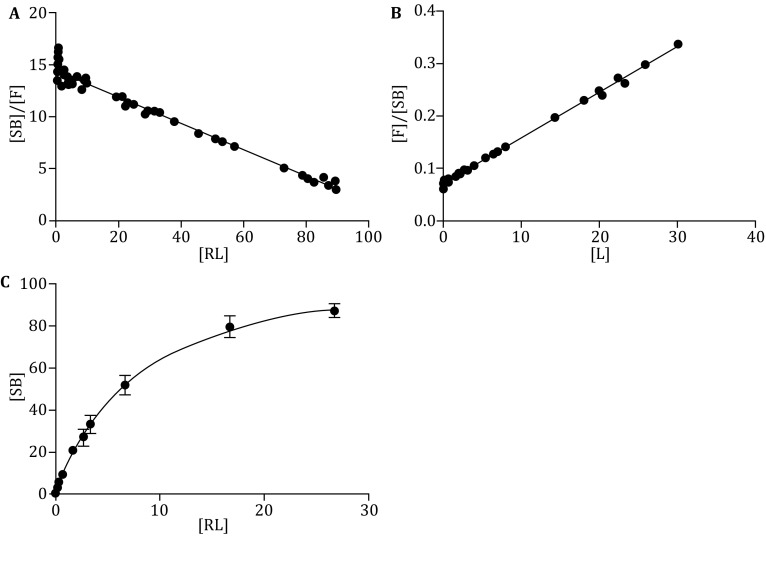
The calculation of *K*_d_ and *B*_max_ by Scatchard plot (A), Woolf plot (B) or the software (C)

Figures 1 and 2 present typical representative data obtained using the method described here. EGFR overexpressing UM-SCC-22B cells were assayed against the radiolabeled antibody ^125^I-Nimotuzumab. The labeling yield of ^125^I-Nimotuzumab was 97.6% and the radiochemical purity was >98.5% after purification (Fig. 1). The specific activity was 24.7 Ci/g.

Figure 2A shows an example of a typical equilibrium saturation curve using the radiolabeled assay with increasing concentrations of ^125^I-Nimotuzumab. Figure 2B shows a typical “Scatchard plot”. Figure 2C shows a typical “Woolf plot”. In parallel comparisons from the same data obtained from the binding assays, the Scatchard plot, the Woolf plot and the saturation radioligand-binding curve gave similar estimates of the *K*_d_ (7.81, 7.935 and 8.095 pL/mol, respectively) and *B*_max_ (113.4, 114.5 and 115.3 pmol/L) (Table 3). The average total number of EGF receptors on each UM-SCC-22B cell could be calculated:

$${\text{EGFRS per cell}} = \frac{{115.3\,{\text{pM}} \times 2 0 0\,{\upmu} {\text{L}} \times 6. 0 2\times 1 0^{23} }}{{1 \times 10^{5} }} = 1.4 \times 10^{5}.$$

**Table 3:** Comparison of the results obtained by three plots

	Scatchard plot	Woolf plot	Saturation binding curve
*K*_d_	7.81	7.935	8.095
*B*_max_	113.4	114.5	115.3

## MATERIALS

### Reagents

- 0.2 mol/L phosphate buffer, pH 7.4 (NaH_2_PO_4_·2H_2_O 0.4063 g, Na_2_HPO_4_·12H_2_O 5.8077 g in 200 mL ddH_2_O)
- Chloramine-T (100 µg in 0.2 mol/L PB)
- Na_2_S_2_O_5_ (200 µg in 100 µL ddH_2_O)
- KI (1 mg in 100 µL ddH_2_O)
- Chloroform
- Iodogen (0.5 µg/µL in chloroform)
- Cell-binding buffer (20 mmol/L Tris, 150 mmol/L NaCl, 2 mmol/L CaCl_2_, 1 mmol/L MnCl_2_, 1 mmol/L MgCl_2_, 1% (*wt*/*vol*) bovine serum albumin; pH 7.4)
- PBS buffer (NaH_2_PO_4_·2H_2_O 0.24 g, Na_2_HPO_4_·12H_2_O 2.901 g and NaCl 8.5 g in 1 L ddH_2_O)
- Receptor-overexpressed cells (see “Reagent setup” section)
- Medium
- Fetal bovine serum
- Na^125^I (Perkin Elmer, Waltham, MA, USA)

### Equipment

- pH paper (Aladdin Inc.)
- PD MidiTrap G-25 column (GE Healthcare, cat. no. 28-9180-08)
- MultiScreen™ Vacuum Manifold 96-well plate (Millipore, cat. no. MAVM0960R)
- Vacuum pump (Zhengzhou Greatwall Inc., SHB-III)
- Dry bath incubator (Fisher Scientific, cat. no. 11-718-2)
- γ-counter (PerkinElmer, 2470 automatic gamma counter)
- Glass-bottomed screw cap vial (Agilent Technologies, cat. no. 5182-0715)
- GraphPad Prism (GraphPad Software Inc.)

### Reagent setup

*Cell sample preparation* Culture receptor-overexpressed cells in corresponding medium under certain conditions. Collect the cells from the flasks or the plates. At least 10^5^ cells are needed for each well. It takes 96 wells to test the binding of one ligand with its receptor. Wash the cell solution with 0.01 mol/L sterile PBS three times. Carefully resuspend the cells in cell binding buffer to a concentration of 2 × 10^6^ cells/mL.

*Cold protein L preparation* 500 mg of protein L is dissolved in or diluted with 2.5 mL cell binding buffer. However, it takes a large amount of ligand to finish the experiment. In general, the cold ligands should be 1000 times more concentrated than the radiolabeled ligand to block the receptors. Fewer cold ligands could be used in low concentrations. Moreover, fewer concentrations (for example, eight concentrations) could conserve many ligands.

### Equipment setup

*γ-counter* *E*% could be determined by comparison between the detected cpm value and the objective cpm value.
